# Nanotechnology-Based Approaches for the Management of Diabetes Mellitus: An Innovative Solution to Long-Lasting Challenges in Antidiabetic Drug Delivery

**DOI:** 10.3390/pharmaceutics16121572

**Published:** 2024-12-09

**Authors:** Shounak Sarkhel, Saikat Mollick Shuvo, Md Ahesan Ansari, Sourav Mondal, Pritam Kapat, Arindam Ghosh, Tanima Sarkar, Ranu Biswas, Leonard Ionut Atanase, Alexandru Carauleanu

**Affiliations:** 1 Department of Pharmaceutical Technology, Jadavpur University, Kolkata 700032, WB, India; shounak2013@gmail.com (S.S.); mdahesanansari@gmail.com (M.A.A.); souravtaki93@gmail.com (S.M.); pritamkapat.pk@gmail.com (P.K.); ghosharindam2114@gmail.com (A.G.); tanimasarkar19@gmail.com (T.S.); 2 Department of Pharmaceutical Technology, JIS University, Agarpara, Kolkata 700109, WB, India; saikatshuvo24@gmail.com; 3 Faculty of Medicine, “Apollonia” University of Iasi, 700511 Iasi, Romania; 4 Academy of Romanian Scientists, 050045 Bucharest, Romania; 5 Department of Obstetrics and Gynecology, Grigore T. Popa University of Medicine and Pharmacy, 700111 Iasi, Romania; drcarauleanu@yahoo.com

**Keywords:** diabetes mellitus, nanocarriers, blood glucose

## Abstract

Diabetes is a widespread metabolic illness. Mismanagement of diabetes can lead to severe complications that tremendously impact patients’ quality of life. The assimilation of nanotechnology in diabetes care holds the potential to revolutionize treatment paradigms, improve patient outcomes, and reduce the economic burden associated with this pervasive disease. This manuscript explores the multifaceted utilization of nanomaterials in diabetes care, emphasizing the unique features of nano-based medication delivery methods and smart drug delivery mechanisms. Additionally, this paper talks about research on nanocarrier-integrated oral, transdermal, and inhalable insulin delivery; dendrimer- and nanocarrier-coupled antisense oligonucleotide-driven gene therapy; the implementation of gold nanoparticles and quantum dots for glucose surveillance; and nucleic acid therapies. There are certain restrictions when using medication delivery methods that are commonly available to handle diabetes. In order to increase efficacy and safety, the rapidly developing science of nanotechnology is also being explored and employed in medical biology. Nanomaterials like liposomes, dendrimers, niosomes, polymeric and metallic nanocarriers, and solid lipid nanoparticles are among the nanocarriers that have been developed for better delivery of various oral hypoglycemic agents in comparison to conventional therapies. These nanocarriers provide great control over elevated blood glucose levels, making them one of the most intriguing and promising technologies available today. Furthermore, adding additional ligands to nanocarriers allows for more focused distribution while protecting the encapsulated hypoglycemic drugs.

## 1. Introduction

Diabetes has emerged as one of the most significant global health and economic burdens worldwide, owing to its increasing prevalence and high complexity ratio. Elevated blood glucose levels arising from either insulin insensitivity, inadequate insulin release, or a blend of both circumstances are the features of diabetes mellitus, a common chronic metabolic illness [1,2]. The proportion of people with diabetes is continuously expanding on a global basis, as indicated by numerous reports illustrating a substantial increase in the number of sufferers.

The World Health Organization (WHO) estimates that approximately 422 million individuals worldwide suffer from diabetes with low–middle-income countries causing a majority of cases, and annually, 1.5 million fatalities are directly associated with diabetes [3]. Diabetes poses a significant public health challenge and necessitates urgent attention in terms of prevention, management, and treatment strategies. Additionally, the International Diabetes Federation (IDF) Diabetes Atlas for 2024 highlights that about 10.5% of adult individuals between 20 and 79 years of age are living with diabetes, and almost 50 percent of them are unaware of the status of their diseases. Moreover, the projections conducted by the IDF anticipated that, by 2045, an estimated 783 million adults, or about 1 in 8 individuals, will have diabetes, reflecting a 46% rise from the present figures [4,5].

Diabetes is an enduring and persistent metabolic condition, provoking increased levels of glucose in the bloodstream [6]. Type I diabetes mellitus stands as an autoimmune variety of diabetes that commonly emerges in young individuals as a consequence of an insufficient supply of insulin. Conversely, type II diabetes mellitus is not insulin-dependent and makes up around 90% of diabetes cases. This type of diabetes typically occurs in adults and is associated with lifestyle factors, such as diet and physical activity [7,8]. Insulin, a hormone secreted by the βcells of the pancreas, plays a crucial role in metabolizing glucose derived from food to produce energy [9]. Persons with type I diabetes have a pancreas that either does not make any insulin at all or makes inadequate amounts of it. Although this kind of diabetes may occur in anyone at any age, it is typically identified in children and young people. To regulate blood glucose levels, daily insulin injections are needed.

In type II diabetes mellitus, the pancreas may not secrete sufficient insulin, or the insulin produced may not be utilized effectively by the liver, muscles, and fat cells, leading to a condition known as resistance to insulin. Because of insulin resistance, cells need more insulin for converting glucose into energy, which inevitably raises blood sugar levels and leads to hyperglycemia [10].

The hallmark features of diabetes mellitus include hyperglycemia, insulin resistance, and a relative deficiency in insulin. The development of type II diabetes mellitus is often influenced by lifestyle choices, genetic predisposition, and environmental and behavioral risk factors [11,12]. Given the progressive nature of the disease, managing and treating type II diabetes mellitus poses a complex and challenging task. Failure to adequately address diabetes can lead to severe complications, underscoring the importance of closely monitoring various parameters such as glucose levels, blood pressure, and lipid profiles to mitigate overall risks in patients with type II diabetes mellitus [13,14]. Insulin therapy remains a cornerstone in dealing with nearly all diabetic-affected individuals by providing the necessary insulin replacement. Mismanagement of the disease like inadequate control and supervision of diabetes mellitus can result in severe and potentially fatal health issues, which include lower-limb amputation, nerve damage (neuropathy), vision impairment, various cardiovascular conditions, and kidney failure [15].

Traditional pharmacological treatments for diabetes, like oral antidiabetic drugs and insulin therapy, often encounter limitations, such as inadequate efficacy due to incorrect dosing, the first pass effect, P-glycoprotein efflux, decreased capability or impacts from drug ingestion, the absence of target particularity, a short duration of action, and adverse reactions, all of which can affect patient adherence and the comprehensive management of the condition [16,17]. Moreover, the conventional therapeutic approaches, although effective in controlling blood sugar levels, frequently prove insufficient in addressing the intricate pathophysiology of diabetes and its complications, thereby prompting the need to explore more advanced therapeutic modalities [18]. In this context, the realm of nanotechnology has emerged as a promising avenue to tackle the challenges linked with traditional diabetes treatments. Nanocarriers possess distinctive physicochemical characteristics that can be harnessed to amplify the delivery and effectiveness of antidiabetic medications [19]. These gradually developed nanocarriers embedded in nanoformulations offer several benefits over traditional formulations, such as heightened intestinal drug penetration, prolonged gastric holding of the drug, inhibition of P-glycoprotein efflux, amplified selectivity, enhanced capability, intended drug distribution, regulated and prolonged drug release, and improved bioavailability, among others [20]. The delivery of insulin through nanocarriers is of particular significance due to its ability to provide a more convenient, secure, and non-invasive approach to administering insulin, ultimately overcoming challenges such as insulin resistance in the management of diabetes. Moreover, the process of encapsulating drugs within nanomaterials has the potential to enhance the stability of the medication by offering protection against enzymatic and chemical breakdown in the gastrointestinal tract (GIT), thereby boosting the medication delivery systems’ overall potency. This inventive strategy has the potential to revolutionize the delivery of drugs via addressing the key issues related to drug efficacy and patient compliance, paving the way for advancements in personalized medicine and targeted therapies [21,22].

The present review addresses numerous nanotechnology-based nanocarriers as conveyors for the usage of medications in the management of diabetes mellitus (DM). Furthermore, the significance of formulating nanomedicines for antidiabetic agents has been underscored as a means to tackle the limitations of conventional treatment methodologies. Consequently, methods for the efficacious delivery of therapeutic medications have entailed the integration of these agents within the milieu of the nanocarriers, along with the objective of precisely targeting them for enhanced efficacy and safety.

All the data and information were collected from Science Direct, SpringerLink, Web of Science, EMBASE, Google Scholar, Scopus, PubMed, Mendeley, and other databases. These data and the information were collected by using the following keywords: nanocarriers, blood glucose, diabetes mellitus, solid lipid nanoparticles, liposomes, niosomes, polymeric nanoparticles, dendrimer-derived nanostructure, silver nanoparticles, gold nanoparticles, quantum dots, metal–organic framework, DNA origami, etc.

## 2. Fundamentals of Nanocarriers

Nanocarriers have surfaced as a favorable strategy in the realm of drug administration, presenting a multitude of benefits when compared to traditional pharmaceutical formulations. These colloidal systems, typically less than 1000 nm in size, have the ability to enhance the pharmacokinetic and pharmacodynamic properties of therapeutic agents, resulting in less adverse effects and greater efficacy [23].

Pharmaceutical nanocarriers can be defined as nanoengineered vehicles being used as a transport module for therapeutic agents, along with having high versatility [24]. In general, there are two types of nanocarriers: organic and inorganic. The organic system includes liposomes, niosomes, lipid nanoparticles, polymeric nanoparticles, dendrimers, micelles, virus-like particles (VLPs), and so on. On the contrary, metallic nanoparticles and mesoporous silica nanoparticles (MSNs) are examples of inorganic nanocarriers [25,26].

Nanocarriers can customize the fundamental features as well as the bioactivity of their enclosed moiety to bring about better pharmacokinetic and biological distribution outlines, diminished toxicity, regulated release, heightened solubility along with stability, and sustained payload delivery. Therefore, the shortcomings associated with traditional medication delivery methods can be overcome by nanocarriers [27].

## 3. Merits of Nanotechnological Inclusion in Diabetic Medications

**Figure 1:**
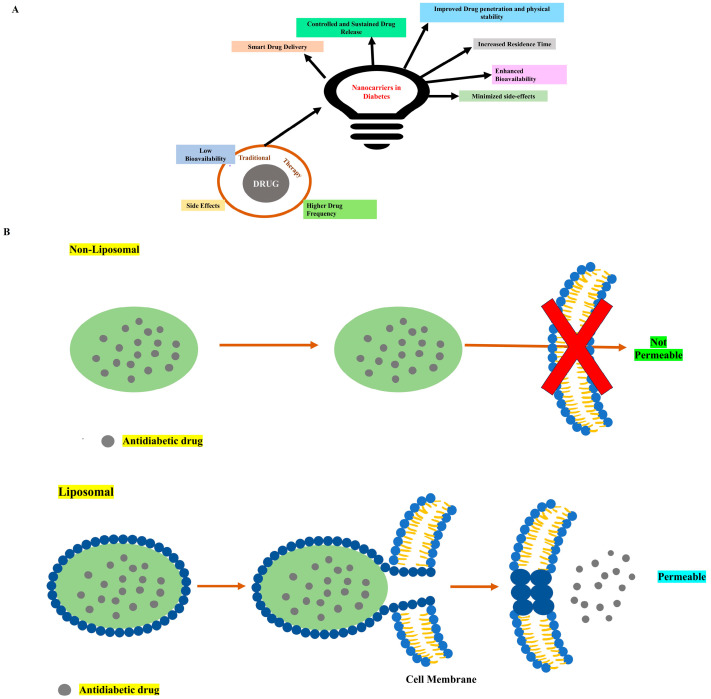
Nanotechnology-based approaches to address challenges associated with traditional therapy. (**A**) Numerous benefits of implementation of nanotechnology in diabetes treatment compared to conventional treatment procedure(drawn by authors). (**B**) Illustration of liposomal vs. non-liposomal antidiabetic drug delivery based on permeability. Here, red “X” indication to show the fact that non-liposomal antidiabetic medications are unable to permeate through the cell membrane. Since the liposomal system is permeable, the encapsulated antidiabetic medications can potentially be transported to the target cells with greater efficiency [31].

Nanotechnology is a novel and cutting-edge strategy for delivering nano-therapeutics in drug delivery to deal with diabetes. Nanotechnology-driven nanocarriers have brought about a revolutionary impact within the realm of pharmaceutical research and development, facilitating precise manipulation of the shape, particle size, surface characteristics, and release of pharmacologically active ingredients at the specific target sites, ensuring a rationalized rate and dosage [24]. Nanocarriers have been prepared using various types of natural, organic, and inorganic materials that belong to polymers, metals, ceramic, and lipids. Pharmacologically active moieties are incorporated into nanocarriers through physical interactions, including entrapment or encapsulation and surface attachment [28]. These variations could certainly be introduced by the unique properties of several nanocarriers that resolve the drawbacks related with conventional drug delivery systems [29]. The treatment of diabetes typically involves the use of insulin, oral hypoglycemic agents, or incretin-based therapies. However, conventional delivery methods face challenges, such as enzymatic breakdown, suboptimal pharmacokinetics, and invasive administration techniques [30]. Nanocarriers offer an innovative approach by providing benefits, such as improved drug stability and absorption, targeted delivery to specific tissues or cells, controlled or stimuli-responsive drug release, enhanced bioavailability, reduced side effects, and better patient adherence (Figure 1A). For instance, liposomal delivery systems excel in enhancing the permeability and cellular uptake of antidiabetic drugs, enabling targeted release and improving therapeutic efficiency. In contrast, non-liposomal carriers often struggle to cross lipid bilayers, limiting their effectiveness in delivering drugs to the target cells (Figure 1B) [31]. Additionally, by facilitating non-invasive insulin administration modalities such as oral or nasal delivery, nanocarriers assist in alleviating injection-related consumer compliance concerns.

## 4. Nanocarriers for Enhanced Delivery of Diabetic Medications

**Figure 2:**
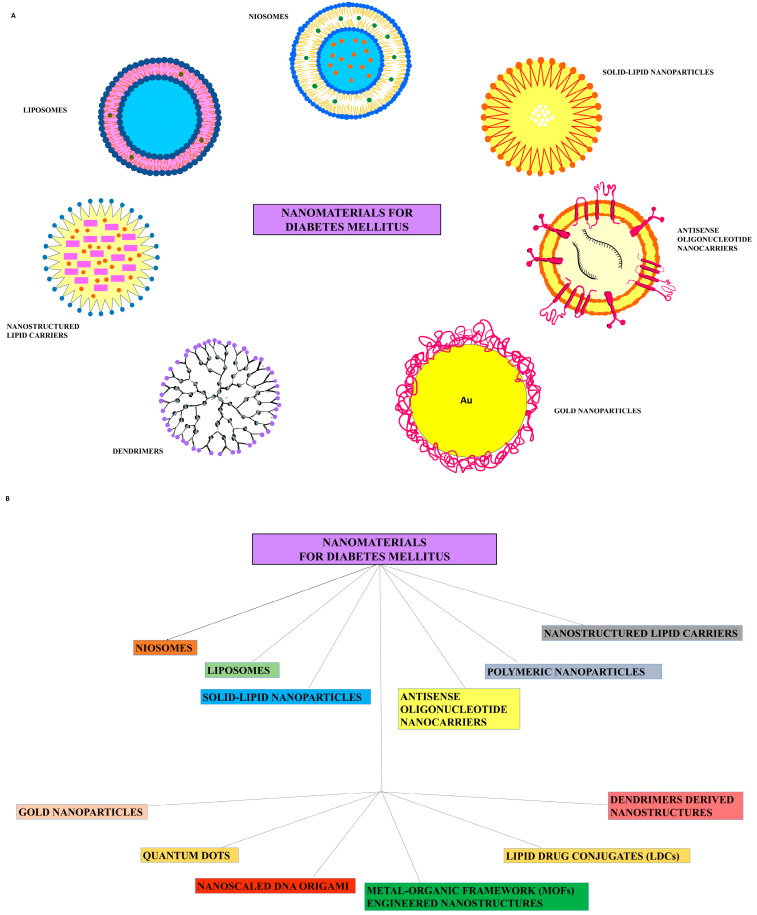
(**A**)Emerging nanomaterials/nanocarriers for treatment and regulation of diabetes mellitus [32]. (**B**)Flow prism of nanomaterials for diabetes mellitus (drawn by authors).

Nanocarriers possess tremendous possibilities in boosting the convenience as well as potency of diabetes medication administration. Their adaptability may assist to connect essential unmet demands for diabetes care, ranging from focused therapy to controlled medication release. A detailed list of certain advanced nanocarrier-based tactics appears below (Figure 2) [32].

### 4.1. Solid Lipid Nanoparticles (SLNs)

SLNs can be designated as proficient pharmaceutical nanocarriers tailored for regulated drug delivery. These are physiological submicron colloidal carriers, which are lipid-based and are dispersed in either water or in water-based surfactant solution. They have a size spectrum spanning from 50 to 1000 nm [32]. Typically, SLNs are characterized by a monolayer of phospholipid coating over a solid hydrophobic core. They represent a novel class of lipid emulsions with a submicron size wherein the solid lipid has substituted the liquid counterpart. They have a unique small size and an expansive surface area, which confer upon them an exceptionally high drug-loading capacity and the capability to incorporate both lipophilic and hydrophilic agents. Medications are usually embedded in amorphous clusters inside crystal fractures or, simultaneously, in the gaps between lipid layers and fatty acid chains. SLNs comprise biocompatible constituents that are physiologically acceptable and homologous to polymeric nanoparticles, just like other carrier systems. Furthermore, the solid matrix of SLNs preserves the entrapped therapeutic molecules from the harsh biological environment and other chemical degradations, enhancing the likelihood that the release patterns of the therapeutic molecule will be modulated with the best possible efficacy [33,34]. In general, SLNs are produced mainly using biodegradable lipids, such as fatty acids (stearic acid and palmitic acid), partial glycerides (glyceryl monostearates and glyceryl palmostearate),and triglycerides (tricarpin and tripalmitin) [35]. Insulin is utilized to deal with insulin-dependent diabetes or type I diabetes mellitus. In that case, insulin is administered through daily injections or an insulin pump, which is painful and uncomfortable. Oral administration of insulin is challenging and ineffective due to the enzymatic activity of protein digestive enzymes, including proteases, pepsins, trypsin, chymotrypsin, and carboxypeptidases, and a certain cytosolic enzyme known as the insulin-degrading enzyme, as well as the physical surroundings of the gastrointestinal tract (GIT). The safeguarding of labile proteins such as insulin from enzymatic breakdown and the harsh surroundings of the gastrointestinal tract (GIT) can be performed through the encapsulation technique, which also facilitates a regulated release pattern [36].

Wang M et al.’s [37] research concentrated on positively and negatively charged solid lipid nanoparticles (SLNs), which were prepared for oral insulin administration by coating the particles surface with positively charged chitosan and negatively charged poly-glutamic acid (PGA), respectively. Through a pharmacodynamic and pharmacokinetic assessment in diabetic rats, their findings indicated that the absorption rate of insulin in the intestine was greatly improved.

Ansari et al. [38] fabricated solid lipid nanoparticles filled with insulin for oral insulin delivery. They demonstrated that the bioavailability of SLN-incorporated insulin was five times higher than that of pure insulin solution (8.26% observed against 1.7%), suggesting enhanced gastrointestinal protection of insulin.

Solid lipid nanoparticles (SLNs) containing glibenclamide (GLB), a drug with inadequate solubility in water, were developed by Elbahwy et al. [39] to achieve regulated and prolonged release and improved oral bioavailability. SLNs were created through employing the ultrasonication approach. In vivo exploration on the histological, pharmacodynamic, and pharmacokinetic properties of optimized SLNs showed reduced blood glucose levels with a short half-life (0.5 h) and a sustained duration of action (24 h). Further, their research revealed that SLNs of GLB profoundly impacted the management of diabetes in a model of diabetic rats.

In addition, in this modern era, pharmaceutical research is seeking alternative routes for insulin administration and the pulmonary route can serve as a potential substitute in this case, which offers several advantages including the extensive absorption area of the lungs, large vasculature, and enhanced permeability of the alveolar epithelium membrane. Liu et al. [40] examined an innovative nebulizer-compatible device for administering insulin-loaded solid lipid nanoparticles (SLNs) via the pulmonary route, which is developed through the micelle–double emulsion method. Characterization parameters such as size and morphology, along with the previous and post-nebulization concentration of insulin, indicated optimum compatibility with this delivery method. Furthermore, the administration of insulin through pulmonary delivery via SLN carriers effectively decreased plasma glucose levels in in vivo testing on rat models (Table 1).

### 4.2. Lipid–Drug Conjugates (LDCs)

Lipid carriers are a worthy option for transporting lipophilic drug molecules and, to a certain degree, hydrophilic drugs [41]. With a great amount of hydrophilic drug-loading capabilities, lipid–drug conjugates (LDCs) have addressed the shortcomings of existing lipid-based nanocarriers, like nanostructured lipid carriers and solid lipid nanoparticles [42].

Nevertheless, hydrophilic medications frequently exhibit suboptimal oral bioavailability owing to their intrinsic hydrophilic traits. The fundamental concept of lipid–drug conjugation involves transforming drugs with hydrophilic properties into a more lipophilic form, thereby transforming them into a less soluble molecule by bonding them with a lipid compound. Consequently, lipid–drug conjugates improve the oral bioavailability of hydrophilic drug prospects [43]. These systems offer several prominent advantages, including safeguarding the drug against degradation and provoking its lipophilicity and the resultant enhanced penetration through the gastrointestinal (GIT) wall. Lipids contained in LDC nanoparticles promote oral absorption [44]. They exhibit inadequate solubility in water and possess a standard melting temperature range of around 50–100 °C. Through the utilization of the High-Pressure Homogenization (HPH) method, these LDCs can be reshaped into nanoparticles. In addition, LDCs can be synthesized by the fusion of innumerable techniques, including salt formation with fatty acids, e.g., the reaction of the functional groups of the drugs with the carboxylic groups of the fatty acids, through direct covalent bonding or covalent linkage, such as ethers or esters [45,46].

Ikumi and colleagues [47] synthesized a poly-glutamic acid derivative customized with phloridzin, an inhibitor of the Na+/glucose co-transporter (SGLT-1), through the use of an α-amino triethylene glycol linkage. The outcomes of the undertaken in vitro and in vivo assessment on rats with diabetes presented that poly-glutamic acid–phloridzin conjugation significantly impeded glucose transport and potently controlled the rise in blood glucose levels following oral glucose administration. On the contrary, unbound phloridzin exhibited negligible effects on hyperglycemia induced by glucose. These findings suggest that poly-glutamic acid–phloridzin conjugation holds promise as a viable agent in oral antidiabetic therapy.

### 4.3. Liposomes

In the 1970s, liposomes were proclaimed as a promising vehicle in drug delivery systems (DDSs). Liposomes, also labeled as phospholipid vesicles, structurally resemble cell membranes, and they represent the characteristics of self-assembled colloidal particles. Primarily, they were employed for studying biological membranes. In modern times, they have evolved into a pivotal model broadly utilized as drug carriers [48,49].

Apparently, liposomes are artificial vesicles with a diameter range from nano- to micrometers, comprising singular or multiple phospholipid bilayers, predominantly phosphatidylcholine, which are tailored from biodegradable, nontoxic, and non-immunogenic phospholipids as well as cholesterol [50]. Liposomes are widely used in both experimental and commercial drug delivery systems owing to their low toxicity, inherent biodegradability, and biocompatibility. These structural arrangements facilitate the entrapment of hydrophilic and lipophilic medications within the vesicle’s aqueous core and its lipid bilayer portion, which is associated with hydrophobicity, or attach to the vesicle’s surface while enabling smart or targeted drug delivery [51,52]. Therefore, magnificent research efforts have been undertaken to alleviate the drug’s toxicity and gain optimum efficacy and safety through targeting particular organs and cells by liposomal formulations. During the lipid material delivery process, liposomes undergo fusion with cellular lipid membranes, subsequently releasing their entire contents into the cell’s cytoplasm to carry out their pharmacological activities [53]. These tiny carriers have garnered significant interest for encapsulating protein- and peptide-classified medications. Several factors must be taken into account to improve the particle size and its efficiency of insulin entrapment within the liposomal core. These factors include the water-to-oil emulsion state ratio, the pH of the buffering agent during hydration, and the phospholipid-to-drug and the phospholipid-to-cholesterol ratios. An appropriate phospholipid-to-cholesterol ratio is crucial for maintaining membrane fluidity, permitting the maximum number of insulin molecules to be incorporated, and inhibiting insulin leakage from the liposomal core [54]. Furthermore, the degree to which drug molecules bind to the liposome surface can be controlled by a few parameters, like temperature, configuration, or the makeup of the lipid bilayers. Progressive research efforts reveal the optimal conditions for insulin attachment with liposomes, which are lower temperatures and small unilamellar liposomes. Through hydrophobic interactions, around 40–60 µg of insulin might attach to 1 µmol of phospholipids [55,56].

Zhang and colleagues [57] explored how insulin can be delivered orally using liposomes modified with the particular ligand biotin (BLPs)and studied their cytotoxicity. BLPs are integrated through supplementing the liposomes’ lipid bilayer with biotin-1,2-distearoyl-sn-glycero-3-phosphatidylethanolamine (DSPE). The particle size along with the entrapment efficiency (EE%) of the liposomes loaded with insulin were found to be impactful throughout the research project. The researchers found that a lipid-to-cholesterol ratio of 3:1 was effective in holding more insulin within the liposomes, providing optimal membrane fluidity and diminishing the likelihood of the inner aqueous segment leaking insulin from the formulated liposomes. Moreover, the amount of biotin-DSPE in the liposomes, their particle size, and the formulation’s dosages showed a significant impact on the hypoglycemic effect. Notably, employing 153.7 nm liposomes brought on a notable hypoglycemic effect, likely due to their enhanced stability and improved uptake via receptor-mediated endocytosis through the intestinal epithelial cells. In addition, the hypoglycemic effect of BLPs showed a linear response at low doses, but at higher doses, the effect became nonlinear owing to the BLPs having strongly saturated the enterocytes’ biotin receptors. The BLPs demonstrated that the pharmacological bioavailability was increased 5.28-fold, when compared with conventional treatment, indicating their potential as effective carriers for oral insulin delivery.

Shafiq et al. [58] synthesized liposomes from camel milk’s fat globule membrane (MFGM) for overcoming the drawbacks of subcutaneous insulin therapy by delivering insulin orally. Their study indicates that the thin-film hydration approach was utilized to construct liposomes. The encapsulation of insulin within the liposomes was confirmed by Fourier transform infrared (FTIR) spectrometric evaluation as evidenced by the changes in their size and charge. In addition, the negative sign of interaction was found through FTIR analysis, which notifies that the drug-excipients are compatible. Using the MTT assay, in vitro cytotoxicity testing on HEK-293 cell lines revealed more than 90% cell viability. Additionally, the effects of the liposomes had been assessed through an in vivo test using a model of diabetic rats induced by streptozotocin (STZ). The study showed that the liposomes considerably lowered the levels of bilirubin, albumin, alanine aminotransferase (ALT), ALP, and blood glucose. According to hepatic histological examinations, all treatment groups exhibited improvement and there were no distinguishable microscopic variations in the kidney tissue.

In another study by Hu et al. [59] for the purpose of administering type 2 diabetes medication, metformin was encapsulated in liposomes modified with hyodeoxycholic acid. Similar to cholesterol in configuration, hyodeoxycholic acid can lower blood glucose levels and control glucose homeostasis. Three different liposome types have been fabricated by the researchers using the thin-film dispersion method, each having a different proportion of metformin and hyodeoxycholic acid, like HDCA: MET (0.5:1), HDCA: MET (1:1), and HDCA: MET (2:1). A greater amount of hyodeoxycholic acid caused a reduced drug-loading percentage. On the basis of in vivo experiments conducted on diabetic mice, the liposomes demonstrated the capacity to minimize the level of blood glucose under fasting conditions, amplify glucose tolerance, modulate markers of oxidative stress, and safeguard the liver tissue. The generated liposome with an HDCA: MET (1:1) ratio was found to be most successful and achieved better results compared to metformin alone. In addition, the enhanced hypoglycemic effect of metformin was observed by utilizing hyodeoxycholic acid as an excipient in the liposome formulation.

Wu et al. [60] synthesized arginine–insulin complexes (AINSs) integrated into liposomes (Lip) that were embedded in a hydrogel consisting of cysteine-modified alginate (Cys-Alg). This helped to mitigate the problems associated with oral insulin delivery, such as early drug release, poor intestinal absorption, and weak adhesion to the intestinal walls, resulting in low treatment efficacy. The AINS-liposomal-gel effectively dropped blood sugar levels and released insulin in a structured manner, as demonstrated by in vivo tests. Additionally, the hydrogel enhanced insulin’s retention on the intestinal mucosa and facilitated its release from the liposomes. Furthermore, an ex vivo experiment revealed that, in comparison to free insulin, the AINS and AINS–liposome conjugation showed nearly 2.0 and 6.0 times higher intestinal penetration, respectively (Table 1).

### 4.4. Niosomes

Niosomes represent a highly promising option for transporting a wide range of pharmacological and diagnostic substances. These are nonionic surfactant vesicles and were originally conceptualized by Ballie et al. back in 1985. Niosomes can be defined as a type of molecular cluster that originated from the self-amalgamation of nonionic surfactants within water-based surroundings [61]. This structure consists of a polar component located on the exterior surface, coupled with a non-polar region internally. These amphiphilic compounds, termed surfactants, possess both hydrophobic (tail) and hydrophilic (head) segments, leading to their ability to self-assemble and form various structures, like micelles or flat lamellar bilayers [62]. Among the surfactants suitable for serving as potential constituents for niosomal drug delivery are sorbitan esters and their derivatives, as well as surfactants based on sugars, polyoxyethylene, polyglycerol, or crown ethers. In some cases, membrane additives like cholesterol or its analogs may also be incorporated into the system. Nonionic surfactants are the preferred choice due to their reduced potential to induce irritation. Niosomes are vesicular structures that may encapsulate both lipophilic and hydrophilic materials because of their distinct topologies. Lipophilic molecules are sequestered by being distributed into the lipophilic region of the lipid bilayer arrangement, while hydrophilic drugs are typically confined within the internal aqueous core or adhere to the bilayer surfaces [63,64].The construction of vesicular structures requires a specified amount of input energy, and all investigated experimental methods involved hydrating a combination of surfactants beyond the gel-to-liquid phase transition temperature, followed by precise size reduction for the creation of colloidal dispersion [65]. These vesicles have been commonly used as a vehicle for drug delivery purposes because of their ability to carry a range of therapeutic substances, with the goals of achieving medication targeting, improved permeation, and regulated release. Indeed, niosomes have the potential to serve as therapeutic reservoirs, facilitating the regulated distribution of medicines to improve their bioavailability and yield enduring therapeutic effects. Moreover, modifications are possible by altering the composition, adjusting the quantity of various additives, and manipulating the surface charge of the components found in vesicles together with membrane supplements [65,66].

Niosomes are favored over conventional liposomes due to their heightened chemical stability, simplified production process, cost-effectiveness, and versatile selection of surfactants [67]. The utilization of niosomes as a plausible vehicle for drug delivery has been extensively researched, encompassing various routes of administration such as pulmonary, ocular, cutaneous, transdermal, vaginal, and oral [68].

Nevertheless, numerous investigations have been carried out to assess the viability of niosomes in facilitating drug delivery specifically for diabetic treatment. A research investigation was carried out by Pardakhty et al. [69] regarding the trapping of insulin within the bilayer structure of niosomes, which leads to protection against the proteolytic effects of trypsin, pepsin, and alpha-chymotrypsin. Based on the in vitro findings, at a molar ratio of 7:3, the brij 92/cholesterol formulation variant exhibited the most impactful protection, with only 26.3 ± 3.98% of the encapsulated insulin. The insulin released over the course of a 24 h period in simulated intestinal fluid (SIF). The Baker and Lonsdale equation most accurately described the release kinetics of most formulations, which also showed a diffusion-controlled delivery system. Therefore, niosomes may be employed as a potential entity to create oral dosage forms that would carry peptides and proteins, such as insulin, together with a sustained release capability.

Niosomes have also shown promise for the vaginal application of insulin. Niosomal nanocarriers have been developed using the lipid phase evaporation method in conjunction with sonication to accomplish this. These nanocarriers consisted of two vesicles having particle diameters of 259.7 ± 33.8 nm and 242.5 ± 20.5 nm, using Span 40 and Span 60, respectively. After the introduction of insulin containing niosomal vesicles through the vaginal route in Wistar rats that were both ovariectomized and induced with diabetes using alloxan to maintain vaginal epithelium thickness, research was conducted on the pharmacokinetics and hypoglycemic effects of insulin. The outcomes demonstrated that insulin-Span 40 and insulin-Span 60 were capable of lowering blood glucose levels to a maximum of 47.49% and 46.66%, respectively, and those reductions continued even six hours after vaginal administration. In addition, in contrast to subcutaneous injection, the two versions showed 9.11% and 8.43% increased bioavailability, respectively. After 24 h, the niosomes’ extended insulin release profile indicates that niosomes may be a potential therapeutic tool for vaginal insulin delivery, allowing for regulated and sustained release characteristics to achieve a noticeable hypoglycemic impact [70].

A study on the oral niosome formulation of embelin constructed using the thin-film hydration process was performed by Alam et al. [71]. The medication’s capacity to avert diabetes in Streptozotocin-induced Wistar rats was the main focus of the research. Embelin is a naturally occurring substance that has been shown to have antidiabetic activity among other pharmacological properties. Embelin offers extra advantages as nanoformulations when combined with niosomes. The formulation underwent thorough characterization, with an antioxidant assessment involving superoxide dismutase (SOD), catalase (CAT), thiobarbituric acid-reactive substances (TBARSs), and glutathione (GSH). The ideal formulation showed a substantial hypoglycemia effect similar to repaglinide. Additionally, significant increases in SOD, CAT, and GSH levels together with a decrease in lipid peroxidation validated the formulation’s antioxidant efficacy. Consequently, the trial findings showed the usefulness of the formulation of embelin-loaded niosomes in the treatment of diabetes.

Samed et al. [72] showed the effectiveness of a metformin hydrochloride (a first-generation biguanide-classified medication with hydrophilic features) and glipizide (categorized as a second-generation sulphonyl urea derivative with accompanying hydrophobic features) combination treatment, encapsulated and issued under observation using hydrogen-bonded niosomes. Employing the thin-film hydration technique, tween 80 and cholesterol self-assembled to create niosomes. Confocal laser scanning microscopy was utilized to observe that the hydrophilic element was present within the niosome structure’s watery center, while the hydrophobic molecule was concentrated mainly in the shell region. The Fourier transform infrared (FTIR) spectrometric examination showed the formation of hydrogen-bonding relations between tween 80 and cholesterol. Glipizide and metformin hydrochloride (metformin HCl) were shown to have encapsulation efficiencies of 67.64% and 58.72%, respectively. The kinetics of the medication release were investigated under varying pH conditions, mimicking environments such as blood plasma pH, cellular endosomes, and gastric conditions, revealing a sustained and controlled release pattern extending up to 12–14 h following an initial linear release phase of 8–10 h.

Prasad and colleagues [73] developed a niosomal transdermal drug delivery system encapsulated in a carbopol-based transgel framework with an objective of boosting pioglitazone (PZ) skin penetration. Applying the quality by design (QbD) process, the produced formulations were optimized with an emphasis on transdermal flux, percentage entrapment, and particle size. The niosomal gel demonstrated a transdermal augmentation that was almost 3.16 times greater than the pioglitazone from the control formulation, corroborated with transdermal flux estimation as well as confocal laser scanning microscopic evaluation. For analyzing the pioglitazone bioavailability of the carbopol transgel to that of the commercially available tablet formulation, a substantial rise (2.26 times) in the bioavailability of the pioglitazone-loaded niosomal transgel was found via an in vivo pharmacokinetic experiment (Table 1).

### 4.5. Polymeric Nanoparticles

Polymeric nanoparticles (PNPs) are frequently formulated through spontaneous sophisticated self-assembly, encompassing therapeutic agents enclosed within the PNP core. In recent times, polymeric nanoparticles (NPs) have attracted a great deal of interest in the field of pharmaceutical research and development owing to their flexibility in drug delivery systems. These systems exhibit diverse size and physiochemical attributes rendering them highly suitable for delivering various types of drugs [74]. The fabrication of PNPs is facile and scalable, offering notable in vivo and storage stability, along with the ability to sustain drug release, thereby positioning them as highly promising vehicles [75]. The selection of an appropriate polymer holds paramount importance in the production of polymeric nanoparticles, encompassing natural polymers like chitosan (polysaccharides), human serum albumin, and sodium alginate. Chitosan, functioning as a cationic polymer, can enhance the cellular absorption of NPs via receptor-mediated endocytosis when modified with specific ligands [76]. Synthetic or natural polymeric substances can regulate insulin release and subsequent pharmacological effects. Insulin-loaded nanoparticles, formulated using biodegradable polymers such as polylactide-co-glycolide (PLGA), polyanhydride, polyalkyl cyanoacrylate (PACA), and PLA, are absorbed by intestinal epithelial cells, facilitating the transport of insulin across the intestinal mucosa. Certain researchers have devised insulin-loaded nanoparticles employing a biodegradable poly-ε-caprolactone (PCL) in combination with a nonbiodegradable acrylic polymer, Eudragit RS100, which is effectively taken up by the gastrointestinal tract [77].

PLGA and PLA possess characteristics that are non-immunogenic, biodegradable, biocompatible, and nontoxic and are thereby approved by the USFDA for pharmaceutical applications, displaying successful uptake involving insulin, Tf, and LDL receptors [78]. Polymeric nanoparticle-loaded antidiabetic medications have displayed immense potential in transforming the diabetes management landscape.

In a study carried out by Sheng et al. [79], insulin was associated with a cell-penetrating peptide-protamine and subsequently encapsulated within mucoadhesive polylactide-co-glycolide (PLGA) nanoparticles that were covered with a chitosan derivative, namely, N-trimethyl chitosan chloride. The findings of the research showed that in diabetic rats, the hypoglycemia impact was prolonged and there was prompt initiation of action. In comparison with subcutaneous insulin administration, the estimated oral insulin delivery system’s bioavailability was 17.98 ± 5.61%. Notably, a substantial enhancement was observed in the oral insulin delivery system with increased bioavailability in the experimental subjects, suggesting improved cell integration with the mucoadhesive NPs in comparison with normal insulin.

In addition, preclinical investigations conducted on the lungs of guinea pigs utilizing PLGA nanospheres loaded with insulin revealed a notable decrease in blood glucose levels, exhibiting an extended duration of action lasting up to 48 h in comparison to insulin in solution [80].

According to a study published in 2020 by Mumuni et al. [81], insulin-containing nanoparticles (NPs) were produced by a self-gelation process using natural polymers, like water-soluble snail mucin and chitosan. In this research, mucins were examined for ionic interactions with chitosan in varying concentrations to form insulin-loaded NPs. The chitosan:mucins ratios employed were 1:1 and 2:1. In addition, the solid surfactants Poloxamer and polyvinyl alcohol were deployed in this nanoparticular framework. The characterization of each system was conducted, alongside the assessment of the in vitro release behavior of insulin in phosphate buffer solutions, followed by the quantification of the hypoglycemic impact in rat models with diabetes. The findings showed a prolonged release manner of enclosed insulin lasting for 8 h. Additionally, trials conducted in a living body exhibited a noteworthy reduction in blood sugar proportions in diabetic-induced rats following the oral intake of insulin-containing PNPs when compared to the administration of free oral insulin solution. Moreover, limited insulin plasma clearance, no hepatic enzyme toxicity, and no loss in cell viability were demonstrated in the pharmacokinetic and toxicity studies. These outcomes suggest that the PNP formulations have favorable biocompatibility.

Behin et al. [82] developed transdermal films containing glipizide, incorporating varying concentrations of chitosan ranging from 0.5% to 2.5% *w*/*v*. A control formulation of glipizide was prepared excluding beta-cyclodextrin. The utilization of 1.5% (*w*/*v*) chitosan facilitated the efficient transdermal absorption of glipizide in rats, ensuring a controlled release of the medication. Specifically, chitosan at 1.5% *w*/*v* resulted in a drug release rate of 96% over a 24 h period, demonstrating the highest drug concentration achieved. Importantly, the formulation exhibited no potential for inducing skin irritation.

Zhang et al. [83] integrated metformin into polymeric microneedles, which were subsequently coated with polydopamine/lauric acid (PDA/LA). Additionally, metformin-loaded microneedles coated with PDA/LA were encapsulated within poly-vinylpyrrolidone (PVP) microneedles. Upon insertion of the metformin-based microneedles into the skin tissue of diabetic rats during the study, a notable antidiabetic effect was observed. The bioavailability of the microneedles containing the responsive drug was calculated to be 95.8 ± 2.7%, as compared to the subcutaneous injection of metformin (Table 1).

Research by Gu et al. [84] on an artificial “closed-loop” arrangement was explored through utilizing a blend of glucose-restricted enzyme and Dextran NPs containing recombinant insulin. Oppositely charged polysaccharides including positively charged chitosan and negatively charged alginate were embedded on these NPs, and electrostatic forces caused the formation of a gel-like nano-network. The average size of the NPs coated with alginate was 293 nm, compared to 340 nm for that of the chitosan-coated nanoparticles. Upon the development of the nano-network (NNS) loaded with insulin, it was administered into mice with diabetes, showcasing a robust cohesive attribute that facilitated effortless shaping and administration of the medication. Moreover, the nano-system-originated drug delivery system exhibited a significant response to changes in glucose levels within the body. In particular, there was a 3.6-fold rise in the insulin release rate from the nanoformulation when exposed to hyperglycemic conditions. The insulin release rate peaked at hyperglycemic levels and gradually decreased thereafter. The authors concluded that the insulin release through the nano-system-originated drug delivery system was glucose-dependent, with high blood glucose levels triggering insulin release from the nano-system-originated gel and low glucose levels in the blood inhibiting it, imitating the biological process of insulin secretion from the βcells within the pancreatic islets.

### 4.6. Dendrimer-Derived Nanostructures

The dendritic framework was initially conceptualized and developed by Vogtle et al. in 1978.These structures were initially termed as “cascade molecules”. Dendrimers represent a distinct category of artificial macromolecules, constructed with branches incrementally added around a central multifunctional nucleus. Each tier of branch points generates a new “generation”. The characteristics of dendrimers largely hinge on the nature of their end functions. They are commonly recognized as a class of three-dimensional soft nanoscopic compounds with a unique monodispersed and uniform molecular arrangement [85,86]. Identified as the most contemporary class of polymers (starburst polymers), these dendrimers differ from conventional oligomers or polymers due to their symmetry, extensive branching, and a high density of functional ends. A diverse range of dendrimers have been produced based on the generations, intricacy, and constituent materials employed, including polyamidoamine (PAMAM), polypropylene-imine (PPI), and polylysine dendrimers, which are particularly valuable for delivering both hydrophobic and hydrophilic therapeutic agents [87]. The adaptability of dendrimers for a wide range of applications, such as drug delivery, gene delivery, antioxidant administration, peptide delivery, smart drug delivery, and biomedical imaging, has been demonstrated by numerous studies [88].

Labieniec-Watala et al. [89] examined the most efficient yet safest way to administer dendrimers and analyzed the efficiency of three distinct PAMAM G4 administration methods in rats to lessen the detrimental consequences of hyperglycemia. Control and diabetic groups, generated by streptozotocin, had PAMAM G4 (0.5 μmol/kg b.w.) administered through the intestine, intragastrically, or subcutaneously to Sprague-Dawley rats for a duration of 60 days. The research results revealed that the most effective ways to lessen the long-term impacts of hyperglycemia were via intraperitoneal and subcutaneous administration of PAMAM G4, with intragastric administration being the least useful. Intraperitoneal administration had the highest occurrence of adverse effects, whereas subcutaneous delivery had the lowest. Although intragastric administration had fewer harmful effects compared to intraperitoneal administration, it also had reduced potential for lowering blood sugar levels. Subcutaneous injection was identified as the most balanced approach, offering moderate toxicity of PAMAM dendrimers and effective reduction in long-term severe hyperglycemia markers in long-term diabetic experimentation (Table 1).

Research was performed on the impact of polyamidoamine (PAMAM) dendrimers on rats’ improved levels of peptide and protein medication lung absorption at different doses [0.1–1.0% (*w*/*v*)] and across generations (G0–G3). An in vivo experimental analysis was carried out to assess the pulmonary uptake of peptide and protein drugs, such as insulin and calcitonin, in rats both with and without PAMAM dendrimers. The outcomes of the study showed a substantial improvement in the pulmonary uptake of calcitonin as well as insulin with PAMAM dendrimers in rats, along with the enhancing effects being influenced by the dendrimer generations. Particularly, the effect on absorption went in the following order: G3 > G2 > G1 > G0. In addition, it was found that the absorption enhancement within the same generation was affected by the concentration of PAMAM dendrimers. When these dendrimers were examined for toxicity on lung tissues, no membrane damage was found. The inclusion of PAMAM dendrimers prompted an affirmative shift in the zeta potentials of the insulin as well as calcitonin solutions, which potentially play a role in the mechanisms responsible for enhancing the pulmonary uptake of these drugs in rat models [90].

Kim PH et al. [91] investigated the effectiveness of treating diabetes with an exendin-4-expressing chimeric plasmid in order to improve incretin-based gene therapy. They obtained this by employing a two-step transcription amplification plasmid technique with a PAMAM dendrimer (PAM-ABP) that was conjugated to arginine by grafting. Owingto their specific molecular structures, dendrimers are becoming more recognized as an essential part in the transport of genes. Exendin-4 is an intravenous agonist of the glucagon-like protein-1 receptor that was recently approved by the FDA. It can be used to regulate food ingestion, stomach emptying, and glucagon secretion without causing any systemic distress and to improve the effects of gluco-regulatory processes by stimulating insulin secretion. Exendin-4 expression was generated in ectopic cells through the PAM-ABP/chimeric DNA combination with greater frequency. This, in turn, raised the cAMP levels in the pancreatic beta cells, which in turn boosted protein kinase K activity and eventually boosted insulin secretion. Consequently, dendrimer-based delivery of the exendin-4 system represents a feasible future avenue for incretin gene therapy improvement.

### 4.7. Silver-Based Nanoparticles (AgNPs)

From ancient cultures, silver has been used historically to cure wounds and illnesses. Considering their special characteristics, silver nanoparticles (AgNPs) have become the most popular metallic nanoparticle in the field of therapeutics. The properties of AgNPs under research exhibit remarkable qualities like conductivity, chemical stability, and catalytic capabilities in addition to noteworthy anti-inflammatory, anti-microbial, and antidiabetic actions [92]. Deploying a silver nanoparticulate system has demonstrated a number of advantageous effects, such as an extensive antidiabetic mechanism, efficiency against infections that are resistant to drugs, and little systemic toxicity [93].

Studies have investigated the use of AgNPs in managing diabetes, with a primary focus on their ability to enhance the delivery of antidiabetic medications. For example, AgNPs can be combined with insulin or other hypoglycemic agents to enhance their stability and availability in the body. Evidence suggests that the utilization of silver nanoparticles can contribute to the enhanced control of glucose levels and the safeguarding of pancreatic beta cells from oxidative stress, which is vital in the management of diabetes [94,95].

With extracts from *Allium sativum* bulbs, Jini et al. [96] developed silver nanoparticles (AgNPs) and examined their effect on blocking starch digestion. The results showed that the nanoparticles generated were spherical in shape, had a uniform dispersion, and ranged in size from 10 to 30 nm. The cytotoxicity analysis revealed that the produced AgNPs were safe for normal cells and exhibited a high level of effectiveness in scavenging free radicals. In addition, the assessment of the antidiabetic qualities in vitro showed that the AgNPs boosted the utilization of glucose, diminished the production of glucose in the liver, inhibited the activity of starch-digesting enzymes such as α-amylase and α-glucosidase, and did not trigger the release of insulin from pancreatic cells. Further, it was found that the AgNPs’ silver atoms interacted with particular α-amylase, α-glucosidase, and insulin amino acid residues by in silico studying (molecular docking) their antidiabetic effects.

In the year 2023, Rehman et al. [97] created silver nanoparticles (AI-AgNPs) utilizing *Azadirachta indica* seed extract through blending with the green synthesis method to determine their potential antidiabetic benefits. The antidiabetic properties of the raw extracts from *Azadirachta indica* seeds and the biosynthesized AI-AgNPs have been studied using alpha-amylase inhibitory assays, glucose adsorption assays, and glucose absorption by yeast cells (Table 1).

The assay findings revealed remarkable dose-dependent activity for the crude extracts and AI-AgNPs. In addition, streptozotocin-induced diabetic mice were employed to assess the antidiabetic effects of AI-AgNPs (10 to 40 mg/kg body weight) for a 30-day duration. The outcomes exhibited a notable drop in blood glucose levels together with the regeneration of pancreatic and liver cells, confirming the potent antidiabetic effects of AI-AgNPs.

Bhavi et al.’s [98] study examined the use of leaf extract (aq.) from the *Syzygiumcumini* (L.) Skeels plant as a reducing agent to help generate silver nanoparticles (AgNPs) in an environmentally benign method for dealing with diabetes. The results showed that the created AgNPs had a silver content of about 43.18% and were spherical, with an average size of 27.5 nm. Along with their antidiabetic and wound-healing properties, these AgNPs showed positive characteristics of 80.08% glucose uptake as well as 83.91% α-amylase inhibition accordingly. In addition, the assessment of cytotoxicity revealed that the AgNPs had good biocompatibility, even at larger dosages, pointing to a low toxicity profile. Further, the AgNPs exhibited comparable efficacy to regular ascorbic acid, with wound-closure percentages of 27.59% and 92.48% at 12 and 24 h after therapy. These findings suggest that *Syzygiumcumini*-mediated AgNPs have a great deal of potential for use in diabetes and diabetic wound-healing therapies.

### 4.8. Gold-Based Nanoparticles

Gold nanoparticles (AuNPs), varying in dimension from 2 to 100 nm and showcasing diverse shapes such as hollow, rod, diamond, sphere, and prism in core-shell or solid structures, demonstrate extraordinary physicochemical features [99]. AuNPs can be used as a therapeutic treatment because biological molecules, especially proteins and molecules containing cysteine and lysine residues, have an intense binding affinity for AuNPs, which can alter their biological roles and structure [100]. It is important to note that gold nanoparticles (AuNPs) are typically regarded as biologically inert, thus making them well suited for in vivo applications [101]. Gold nanoparticles (AuNPs) are recognized for their stability, biocompatibility, biodegradability, and efficacy and are widely utilized for biomedical purposes, diagnostic imaging systems, sensing, and labeling. However, a number of in vivo investigations have emphasized AuNPs’ antioxidative and antihyperglycemic features [99].

AuNPs are designed particularly to measure patients’ blood glucose levels. Nair and Sreenivasan created a non-enzymatic colorimetric glucose measurement tool with stabilized AuNPs from β-cyclodextrin (βCD) and cyanophenyl boronic acid (CPBA). A green one-pot tandem process was used to make these glucose sensors, which measure the amount of glucose in human blood serum. The biosensors’ sensitivity, functionality, and reaction time were improved by gold-associated nanomaterials [102]. Newly developed biosensors based on AuNPs serve as a diagnostic as well as theranostic tool, integrating nanomaterials into lab-on-chip platforms.

Nowadays, glucose detection in vitro and in vivo is being addressed by nano-biosensor methods. Several glucose monitoring biosensors are sold commercially. These encompass the Abbott FreeStyle Lite, LifeScan One Touch Ultra2, Roche’s Accu-Chek, and Bayer’s Contour. Electrochemical glucose biosensors track the oxidative current generated by the glucose oxidase and identify electric signals that are directly correlated with the quantity of glucose [103].

Omolaja et al. [104] developed and manufactured chalcone-capped AuNPs of *Helichrysum foetidum* plant extract, a South African medicinal herb. According to the study, green synthetic gold nanoparticles of *Helichrysum foetidum* blocked enzymes that hydrolyze carbohydrates and significantly improved glucose absorption in mammalian kidney cells. Additionally, it provided a clinical assessment of the conjugate AuNPs/chalcones with respect to the production of diabetic drugs derived from *H. foetidum* and its gold nanoparticles (Table 1).

Ayyoub et al. [105] investigated the in vivo antidiabetic effects of gold nanoparticles (AuNPs) synthesized using *Dittrichia viscosa* leaf extract in high-fat diet (HFD) and streptozotocin-induced diabetic rats. The study measured the glucose levels and hepatic gene expression of phosphoenolpyruvate carboxykinase (PEPCK), a key enzyme in liver gluconeogenesis. The AuNPs, spherical in shape with sizes ranging from 20 to 50 nm, demonstrated good stability and dispersity as confirmed by the zeta potential and dynamic light scattering (DLS) measurements. The results indicated that these AuNPs could alleviate hyperglycemia in HFD/STZ-induced diabetic rats, potentially by inhibiting hepatic gluconeogenesis through the downregulation of PEPCK gene expression and activity.

In the research carried out by Opris et al. [106], the researchers designed and synthesized gold nanoparticle-integrated *Sambucus nigra* L. (SN) plant extract to assess its antidiabetic potential in a rat model of streptozotocin-induced diabetes. The produced AuNPs, with an average size of 21 nm, were examined for their ability to modulate various parameters associated with diabetes. The findings demonstrated an increase in the antioxidant capacity in the blood, liver, and muscle tissues. Additionally, these nanoparticles effectively lowered blood glucose levels and mitigated inflammation and oxidative stress associated with hyperglycemia.

Another investigation was conducted by Barath ManiKanth et al. [107], which examined the effects of biologically synthesized gold nanoparticles (AuNPs) on diabetic mice produced with streptozotocin. Based on their report, the 50 nm AuNPs, which are generated through the reduction of AuCl_4_-ions via Bacillus licheniformis, exhibited antioxidative qualities that prevent reactive oxygen species (ROS) from being created and scavenge free radicals. The research found that these AuNPs had antihyperglycemic properties, increased the activity of antioxidant defense enzymes, stimulated the regeneration of pancreatic β cells, and lowered blood glucose levels.

### 4.9. Antisense Oligonucleotide-Coupled Nanocarriers

Antisense oligonucleotides (ASOs) are brief, chemically manufactured DNA molecules that have the ability to modify RNA and alter protein expression by various mechanisms. ASOs can hinder gene expression, leading to therapeutic effects. They are essential to the development of antidiabetic medication by regulating the RNA and protein levels of disease-causing gene expression. ASO delivery and targeting are accomplished by the use of nanocarriers. Applying ASOs, siRNA, or aptamers to prevent protein expression via various intracellular pathways is known as oligonucleotide therapy. ASOs can be utilized as potent antidiabetic medicines with improved delivery potency by adopting nanotechnology [108]. ASOs, as genetic materials, display promise in regulating the undesired expression of genes in diseased circumstances by specifically inhibiting the expression of their mRNA targets. ASOs are distributed utilizing a variety of nanotechnology-blended vehicles, including liposomes, dendrimers, protein or peptide conjugates, and carbon nanotubes [109] (Table 1).

Research is being conducted on combining ASO drugs with nanoparticles to identify genes associated with insulin resistance and hyperglycemia. Therapeutic oligonucleotides assist in identifying, functionalizing, and authenticating the genes involved in the pathophysiology of diabetes by applying the gene expression suppression that ASOs generate to inhibit the production of target proteins or RNA levels. Several types of small-molecule ASO medicines have been permitted for the regulation of diabetes type II. The SGLT II inhibitory-classified antidiabetic drug is one that has been approved (e.g., Sotagliflozin, Empagliflozin, Dapagliflozin, Ertugliflozin, and Canagliflozin), as well as alpha-glucosidase inhibitors (e.g., Acarbose and Miglitol) and Dipeptidyl-peptidase 4 inhibitors (e.g., Sitagliptin, Vildagliptin, Saxagliptin, and Linagliptin). Additionally, various antisense-based drugs like ISIS 325568, ISIS 449884, ISIS 113715, and Volanesorsen (ISIS 308401) are currently undergoing clinical trials at different stages [109,110].

### 4.10. Quantum Dots

Quantum dots (QDs) are a type of novel zero-dimensional semiconductor nanoparticle that exhibit fluorescence. These nano-sized particles, which have gained significant attention from researchers over the last two decades, possess intriguing physical and chemical characteristics. Typically, quantum dots consist of a core material enveloped by a shell with a diameter varying from 2 to 10 nm, made from a different semiconductor material [111]. Zinc oxide (ZnO), silica, cadmium (Cd), selenium (Se), and other heavy or organic materials compose the outer shell of these quantum dots. For the purpose of giving a specific location for conjugation and minimizing their toxicity, these components are wrapped in a shell material. The properties of quantum dots, such as optical properties, absorbance, and photoluminescence, are dependent on their size, making them ideal candidates for biological applications and imaging purposes. Their unique attributes position them as promising tools for imaging, sensing, tracking, and real-time monitoring, particularly in the realm of biological sensors [112,113].

Due to their zero-dimensional nature, quantum dots find extensive utility in biological sensors, as demonstrated by Liao et al. [114] in the development of a fiber-optic glucose sensor incorporating ConA (concanavalin A protein)-functionalized QDs. This sensor can be discretely inserted under the skin to monitor the interstitial fluid’s glucose levels continuously because of its flexible and hair-like nature. A fluorescence shift corresponding to the concentration of glucose is caused by the free diffusion of glucose into the sensor and its binding to ConA. Over a period of up to 7 weeks in vitro, the sensor proved effective in quickly and precisely detecting changes in glucose levels (0 to 500 mg/dL detectable range of glucose) in physiological solutions. In a more recent advancement, amino-functionalized silicon quantum dots have demonstrated potential as efficient glucose sensors, showing intensity changes in diluted human blood samples that are proportional to the concentration of glucose while maintaining high particularity for glucose over sucrose and Na+ [115]. However, the sensitivity of the amino-functionalized QDs appears encouraging, and challenges like the limited detection range of sensors restricted its medicinal use owing to the requirement for diluted blood samples. More technological advances, perhaps driven by micro/nanofluidic platforms, might be required to address these challenges in order to enable automated sample pre-processing, such as blood dilution for reliable glucose fluctuation monitoring.

Quantum dots are often likened to artificial atoms due to their electron and atom-like behavior, showcasing movement in three dimensions [111]. Quantum dots such as carbon, graphene, and zinc oxide are preferred for use in applications involving the administration of medications owing to their aqueous solubility and biocompatibility. For example, mitomycin, an anticancer drug, is particularly well suited for delivery by carbon quantum dots. The drug is adsorbed on the surface rather than encapsulated within the quantum dots because of the stiff structure of the dots. This makes it possible for the drug to bind through available functional groups, such as free amine groups (NH2) and carboxylic acid groups (COOH) [116,117].

Efforts in leveraging the drug-binding capabilities of quantum dots demonstrate their potential in targeted drug delivery systems, showcasing a promising avenue for prospective studies and advancements in the area of nanomedicine for diabetes management. Silver sulfide (Ag_2_S) quantum dots (QDs) have the ability to be ingested orally, enhancing the rate at which metformin and nicotinamide mononucleotide is absorbed through the digestive system into the body. The oral bioavailability and targeting of these drugs toward hepatocytes are significantly improved by the incorporation of QDs into their formulation. Although metformin and nicotinamide mononucleotide are already capable of being absorbed orally, their effectiveness is greatly enhanced when combined with QDs, which lead to a rise of 100–10,000 times in potency and overcoming the diminished efficacy associated with aging [118] (Table 1).

However, it is crucial for nanomaterial transporters to exhibit minimal impact on biological systems and to be either naturally eliminated or easily removed from the body post drug delivery [119]. One drawback of using QDs is their tendency to accumulate in the liver or be engulfed by immune cells, potentially causing prolonged exposure and toxicity concerns. Recent advancements aimed at mitigating these toxicity issues involve the application of specific biomimetic coatings and the utilization of nontoxic substances, like silver [120,121].

### 4.11. Metal–Organic Framework (MOF)-Engineered Nanostructures

Metal–organic frameworks (MOFs) provide a dynamic and innovative platform for addressing important biological research issues. These crystalline solids have a very porous structure because they are made up of metal ions or clusters connected by organic ligands. MOF networks can be one-, two-, or three-dimensional and are made up of organic ligands (such as carboxylates, phosphonates, imidazolates, and phenolates) as the struts and clusters of inorganic metals (such as lanthanide or transition metals) as the nodes [122]. MOFs are extremely well designed, with vast surface areas, controlled morphologies, reactivity to stimuli, and well-defined pores [123]. In this regard, a few noteworthy investigations have been documented in the past couple of decades. The synthesis of MOF-5, which is made up of Zn_4_O and 1,4-benzenedicarboxylate (BDC) clusters, was reported by the Yaghi group in the year 1999. The MOF-5 configuration exhibited a broad Langmuir surface area of 2900 m^2^ g^−1^ [124]. In another study, H. Su and associates examined medi-MOF-1, a very porous substance composed of zinc and curcumin intended for the administration of ibuprofen (Ibu). This substance revealed good cytotoxicity and biodegradability characteristics [125]. Moreover, a chiral Zn-based metal–organic framework (MOF) with a high drug-loading capacity and a progressive release of 5-fluorouracil (5-FU) was created for drug delivery [126]. After the MOF particles were shrunk to the nanoscale, the resultant nano-MOFs (NMOFs) may function as efficient nanocarriers for delivering drugs used in photothermal treatment, chemotherapy, imaging, or photodynamic therapy [127]. Compared to other porous materials, MOFs have a number of vital advantages, such as a large surface area and porosity that allow for high loading capacities for therapeutic agents and the simplicity with which inorganic clusters and/or organic ligands may be used to alter their physical and chemical features (such as pore size and shape). For example, when exposed to UV light, some MOFs that include lanthanide metals can glow [128,129]. Furthermore, either pre-design or post-synthetic modification techniques can be used to add functional groups to the organic ligands. Additional advantages of MOFs include the ability of the open pores and windows of the MOF to allow substrates to diffuse and interact with the encapsulated molecules and the moderate strength of the coordination bonds, which contributes to the biodegradability of MOFs as well as their distinct structures, which are advantageous for research on host–guest interactions [130].

MOFs are thought to be the influential option for delivering antidiabetic drugs including insulin, metformin, and GLP-1 analogs because of their unique properties. For instance, MOFs modified with stimuli-responsive linkers can mimic the body’s natural regulatory system by releasing insulin in reaction to variations in glucose levels. Resultantly, several MOF-based electrochemiluminescence (ECL) biosensors, including blood glucose sensors, as well as sensors that detect acetone and isopropanol and additional sensors, have been developed for the diagnosis of diabetes [131]. By employing a surface adhesion approach, Zhang et al. created a new lanthanide-functionalized metal–organic framework enzyme (L-MOF-enzyme) complex by combining Eu3+@UMOF with glucose oxidase (GOx) [132]. When glucose (Glu) was present, Eu3+UMOF acted as a luminous center and as a support for fixation as well as a catalyst for the cascade reaction that produced H_2_O_2_. With a detection limit of roughly 0.2 µM, this MOF-based enzyme complex demonstrated improved stability, excellent fluorescence selectivity, and sensitivity to glucose. Many kinds of gas sensors have been developed recently, including MOF-based sensors for the detection of isopropanol and acetone [130]. Isopropanol (IPA) and acetone are becoming recognized as possible biomarkers for the diagnosis of diabetes. However, it is difficult to measure them directly because of their low amounts in human breath. Therefore, effective procedures for acetone and IPA enrichment are necessary prior to completing analytical processes. For this objective, Yu et al. selected three distinct metal–organic framework types asadsorbents: MOF-5, UiO-66, and ZIF-7 [133]. The quantities of ACE and IPA in actual breath samples were successfully detected using UiO-66, an efficient adsorbent that exhibited great recovery and excellent sensitivity.

MOFs have drawn interest as an efficient platform for insulin administration because of their large surface area, water resilience, and rigid frameworks with tunable mesoporous architectures. They facilitate greater drug loading and significantly improve the encapsulated proteins’ chemical and thermal stability [134,135]. Zhou et al. fabricated a new nanocomposite carrier (Ins-MIL100/SDS-MS) for the oral delivery of insulin (INS) using iron-based MOF nanoparticles (MIL-100). This carrier had a high loading capacity of biodegradable microspheres and exhibited exceptional stability throughout the gastrointestinal system [136]. He et al. developed a “closed-loop” technique for intravenous insulin delivery by encasing a “core-shell” MOF in erythrocyte membranes. The dynamic behavior of the β cells in the pancreas and the glucose-responsive release ofinsulin were intended to be replicated by this technology [137]. To improve the design of nano-MOFs, evaluate their long-term safety, and convert these encouraging findings into practical applications for better diabetes control, further investigation is needed (Table 1).

### 4.12. Nanoscaled DNA Origami

DNA origami is a state-of-the-art nanotechnology technique that creates intricate and accurate 2D and 3D nanostructures through utilizing the special qualities of DNA molecules. This approach led to the creation of several DNA nanostructures by joining the branching DNA junctions, including truncated octahedrons, Borromean rings, and 3D DNA cubes. In 2006, Paul Rothemund introduced the idea of DNA origami, which has since become a significant advancement in the domains of nanotechnology and nanoscience [138,139]. Comparing DNA origami to traditional nanomedicines reveals a number of benefits. DNA origami’s crucial characteristic is the way it is synthesized, which gives complete control over its size and shape. Additional advantages include its stability, programmability, targetability, and biocompatibility, which might make it a practical approach for a variety of biomedical applications [140,141].

DNA origami is a powerful tool that can be used to solve problems with diabetes. These nanostructures have been used for the nanoscale organization of ligands to control receptor-mediated EPH, PD-1, MET, TLR9, FcγR, and B-cell and T-cell receptor signaling events [142,143,144,145,146]. One of the main benefits of DNA origami is its ease of changing the ligand valency, allowing for the fine-tuning of avidity effects between ligand nanoclusters and receptor nanodomains. Scientists from Sweden and Italy have created DNA-insulin nanoclusters, which can regulate insulin signaling in cells. By coming up with insulin molecules on DNA nanorods, they showed control over insulin–receptor interactions and functioning. They suggest that this method may be applied to develop more focused and effective diabetic therapies. One-to-fifteen insulin molecules were affixed at regular intervals to a 140 nm long folded DNA-insulin rod. In a zebrafish embryo model of type 1 diabetes, where the majority of the insulin-producing cells were killed, the researchers also evaluated the nanorods. They discovered that administering multivalent insulin nanorods induced the intended effect, which was an increase in glucose absorption. In contrast, there was no reaction to treatment with nanorods containing only one insulin molecule [147] (Table 1).

**Table 1:** Nanocarrier-incorporated drug delivery systems for treatment and management of diabetes mellitus.

Nanocarrier Systems Used	Antidiabetic Drugs/Entity	Administration’s Route	Nanostructure Particularity	Ref.
Solid Lipid Nanoparticles (SLNs)	Insulin	Oral route	Bioavailability enhanced fivefold compared to the pure insulin solution.	[38]
	Glibenclamide (GLB)	Oral route	Enhanced bioavailability through controlled and sustained release.	[39]
	Insulin	Pulmonary route	Developing an alternative method for insulin delivery with improved effectiveness.	[40]
Liposomes	Insulin (camel milk-derived liposomes)	Oral route	Effective reductionin blood glucose level.	[58]
	Metformin Hcl (hyodeoxycholic acid-modified liposomes)	Oral route	Improved blood sugar-lowering effect of metformin.	[59]
	Insulin (liposomal hydrogel)	Oral route	Enhanced insulin retention duration and intestinal absorption.	[60]
	Insulin	Oral route	Substantially reduced elevated glucose levels and enhanced the drug’s bioavailability.	[57]
Niosomes	Insulin	Vaginal route	Prolonged release of insulin with a significant hypoglycemic effect.	[70]
	Glipizide and metformin hydrochloride	Oral route	Establishing combination therapy and sustain as well as control release pattern.	[72]
	Pioglitazone (Niosomal gel)	Transdermal route	Increased bioavailability compared to marketed pioglitazone tablet.	[73]
Polymeric Nanoparticles	Insulin (PLGA-coated NPs)	Oral route	Enhanced bioavailability with sustained hypoglycemic effects.	[79]
	Insulin (PLGA nanospheres)	Pulmonary route	Reduced blood glucose levels with prolonged duration of action.	[80]
	Glipizide (chitosan coated)	Transdermal route	Higher drug concentration achieved without causing skin irritation.	[82]
	Metformin Hcl (polymeric microneedles)	Dermally inserted	Bioavailability was found to be 95.8 ± 2.7% compared to subcutaneous injection of metformin.	[83]
Dendrimer-Derived Nanostructures	Incretin-based gene therapy	Intravenous route	Increased insulin production.	[91]
	Insulin (PAMAM dendrimers)	Pulmonary route	Improved pulmonary absorption of insulin with the G3 generation.	[90]
	Insulin	Intraperitoneal, intragastric, or subcutaneous routes	Subcutaneous route was identified as the most balanced approach, offering moderate toxicity while lowering hyperglycemia markers.	[89]
Silver Nanoparticles (AgNps)	*Allium sativum* plant extract	Oral route	Enhanced glucose utilization and inhibited starch-digesting enzyme activity without triggering insulin release.	[96]
	*Azadirachta indica* seeds	Oral route	Potential decrease in blood sugar levels and significant dose-dependent activity.	[97]
Gold Nanoparticles (AuNps)	*Helichrysum foetidum* Plant extract (chalcone-capped AuNps)	Oral route	Enhanced glucose uptake in mammalian kidney cells.	[104]
	non-enzymatic colorimetric glucose estimation device	Dermally inserted	Improved sensitivity and faster response time of the biosensor.	[102]
Antisense Oligonucleotide-Coupled Nanocarriers	Gene therapy	Intravenous route	Regulating the expression of genes associated with diabetes mellitus at both the RNA and protein levels, incorporating enhanced potencyand precision-targeted drug delivery mechanisms.	[109]
Quantum Dots	Metformin and nicotinamide mononucleotide	Oral route	Enhanced the drug’s potency by 100 to 10,000 times.	[118]
MOF Nanostructures	Insulin	Intravenous route	Dynamic behaviour of beta cells in pancreas and glucose-responsive release of insulin were replicated by this closed-loop technology.	[137]
Nanoscaled DNA Origami	Insulin receptors	-	DNA origami-integrated insulin nanorods stimulated glucose uptake along with desired response in type I diabetes-prompted zebrafish model.	[147]

Advanced glycation end products (AGEs) are caused by excessive glucose and protein synthesis in a hyperglycemic environment, which accumulate within the body and lead to the generation of ROS and the advancement of the illness. Tetrahedral framework nucleic acids (tFNAs) have the capability to activate the protein kinase B (Akt)/nuclear factor erythroid 2-related factor 2 (Nrf2)/haeme oxygenase-1 (HO-1) signaling pathway in type I diabetic animal models [148]. Vascular endothelial growth factor (VEGF) signaling depends on Akt, a key downstream kinase, whose activation causes neo-vascularization and increased VEGF gene production [149]. Currently, RNA nanotechnology mostly uses nucleic acid nanoparticles to reduce symptoms of diabetic neuropathy as they have been shown to be crucial in its therapy. The inflammatory response can be effectively lowered by miR-146a-5p, which is expressed in varied amounts in the sciatic nerve of T2DM and diabetic neuropathy mice [150,151].

## 5. Clinical Outlook, Obstacles to Clinical Translation, and Future Possibilities

The transition of nanocarriers from research to practical clinical use for diabetes management presents various prospects. Lipid-based nanoparticles, including solid lipid nanoparticles (SLNs) and nanostructured lipid carriers (NLCs), are being designed to enhance the oral bioavailability of antidiabetic drugs with poor solubility, such as glibenclamide. These formulations have shown enhanced pharmacokinetic profiles and sustained drug release, both of which are essential for effective diabetes control [39,152]. Polymeric nanoparticles are frequently explored for oral insulin delivery. Polymers like chitosan, alginate, and poly (lactic-co-glycolic acid) (PLGA) have proven effective in protecting insulin from gastrointestinal degradation, promoting its absorption. Research indicates that these nanoparticles significantly improve insulin bioavailability compared to conventional delivery methods [81,153]. Additionally, nanocarriers designed for gene therapy, such as those coupled with antisense oligonucleotides, offer promising applications. They can deliver oligonucleotides that either stimulate insulin production or regulate immune responses, providing innovative solutions for the long-term management of type 1 diabetes [10]. Functionalized gold nanoparticles (AuNPs) with incretin analogs are also in preclinical stages, targeting pancreatic beta cells to enhance therapeutic outcomes [104]. Encouragingly, FDA-approved nanotechnology-based formulations and pulmonary insulin delivery systems have demonstrated clinical success, highlighting the potential of nanotechnology in diabetes care [154].

Despite the significant potential that nanomaterials hold, there exist a multitude of challenges that must be effectively addressed to ensure their successful utilization in the realm of antidiabetic drug delivery. These challenges encompass various issues such as toxicity, a limited shelf-life, pharmacokinetic properties, stability concerns, costly formulations, diminished entrapment efficiency, as well as challenges related to scalability and manufacturing processes, among others. In order to overcome these obstacles and fully capitalize on the capabilities of nanomaterials in drug delivery systems tailored for antidiabetic treatment, it is imperative to persist in conducting research aimed at creating biocompatible, stable, and scalable nanoformulations [121,154]. In that case, the evolvement in the targeted delivery of antidiabetic drug formulations through the use of nanoformulations, along with the integration of algorithms for artificial intelligence and machine learning to optimize the design, manufacturing, and overall effectiveness of nanotechnology-driven antidiabetic drug delivery methods, is of crucial importance [155]. While nanomaterials have undeniably revolutionized the landscape of diabetes management, there remain numerous areas that hold promise for further advancements, particularly in the realms of biosensor design, medicinal nano-devices, nano-diagnostic tools, and nano-therapeutics. Notably, nanoparticles have exhibited the ability to effectively transport proteins and genes, thereby enhancing therapeutic outcomes significantly [156]. Of particular interest is the potential of nanomaterials in the domain of gene therapy, representing a promising avenue for future exploration and development. In light of these prospects, there is a pressing need to devise simplified and cost-efficient methodologies for fabricating nanomaterials boasting diverse functionalities and morphologies to cater to the evolving demands of the field [152]. As well, a viable remedy may be found through research into and recognition of novel glucose-responsive moieties with greater association constants and materials that include these binding domains [157]. Therefore, ongoing research may pave the way for the formulation of groundbreaking diabetes treatments and address complications linked to diabetes mismanagement.

## 6. Conclusions

To cope with the continual hurdles with handling diabetes mellitus, nanotechnology has appeared as a game-changing strategy. The investigation into nanotechnology and its potential applications in drug delivery has been meticulously researched by many scholars for several decades. Nanomaterials have displayed considerable potential in advancing the delivery of antidiabetic medications, as well as in the realms of diagnosis, glucose sensing, and glucose monitoring. Innovations of nanotechnology-based infrastructures encompassing liposomes, solid lipid nanoparticles (SLNs), polymeric nanoparticles, nanostructured MOFs, quantum dots, DNA origami, and so on, each offer a multitude of benefits, including enhanced bioavailability, targeted delivery, controlled release, improved stability, dose proportionality, reduced toxicity, and decreased dosing frequency. The versatility of the abovementioned nanocarriers allows for the administration of remedies via oral, parenteral, pulmonary, transdermal, and dermal routes, thereby promoting patient adherence to treatment regimens and eliminating the issues associated with dissatisfying injection procedures. In addition, nanoparticles like antisense oligonucleotide-coupled nanocarriers and nanoscaled DNA origami have a lot of promise as routes of administration for nucleic acid therapies, and advancements are probably going to come from better cellular targeting techniques. Due to their minute size, nanoparticles can permeate minor capillaries and be internalized by cells, facilitating effective drug accumulation at specific target sites. These exceptional attributes of nanomaterials offer alternative strategies besides the limitations of conventional antidiabetic therapy administration procedures, optimizing patient management of diabetes in the process. The functionalization of silver and gold nanoparticles with diverse compounds such as molecules, plant extracts, and other entities has been proven to enhance the therapeutic efficacy of antidiabetic medications. Ongoing studies are being conducted to determine whether using nanotechnology for drug delivery is safe in the long run. As a result, the Food and Drug Administration (FDA) of the United States has given out directives to strengthen the safety protocols governing the development of nanotechnology-based products for clinical utilization. In essence, the transition of nanotechnologies from fundamental research endeavors to tangible clinical applications necessitates concerted collaboration among experts from various scientific disciplines. The convergence of interdisciplinary expertise is imperative for catalyzing the transformation of innovative concepts from laboratory settings into commercially viable medical products. By addressing contemporary challenges and capitalizing on forthcoming advancements, nanotechnology holds immense potential for revolutionizing the management of diabetes mellitus and elevating the quality of life for individuals affected by the condition.

## Data Availability

Not applicable.
